# The Evolving Scenario of COVID-19 in Hemodialysis Patients

**DOI:** 10.3390/ijerph191710836

**Published:** 2022-08-31

**Authors:** Pasquale Esposito, Daniela Picciotto, Francesca Cappadona, Elisa Russo, Valeria Falqui, Novella Evelina Conti, Angelica Parodi, Laura Mallia, Sara Cavagnaro, Yuri Battaglia, Francesca Viazzi

**Affiliations:** 1 Department of Internal Medicine, University of Genoa, 16132 Genoa, Italy; 2 Unit of Nephrology, IRCCS Ospedale Policlinico San Martino, 16132 Genova, Italy; 3 Department of Medicine, University of Verona, 37129 Verona, Italy; 4 Nephrology and Dialysis Unit, Pederzoli Hospital, 37019 Peschiera del Garda, Italy

**Keywords:** COVID-19, hemodialysis, mortality, SARS-CoV-2, vaccination

## Abstract

Coronavirus disease 2019 (COVID-19) is a rapidly changing disease. Therefore, in this study, to evaluate the evolution of COVID-19 in hemodialysis patients, we retrospectively compared patients affected by COVID-19 during the first pandemic waves of 2020 (from March to December 2020—Group 1) with patients with COVID-19 from September 2021 to February 2022 (Group 2) after the full completion of vaccination. Group 1 was constituted of 44 patients (69.3 ± 14.6 years), and Group 2 of 55 patients (67.4 ± 15.3 years). Among Group 2, 52 patients (95%) were vaccinated. Patients of Group 2, compared with Group 1, were more often asymptomatic (38 vs. 10%, *p* = 0.002) and reported less frequent fever and pulmonary involvement. At diagnosis, Group 2 showed a significantly higher number of lymphocytes and lower levels of circulating IL-6 (16 ± 13.3 vs. 41 ± 39.4 pg/mL, *p* = 0.002). Moreover, in Group 2, inflammatory parameters significantly improved after a few days from diagnosis. Patients of Group 2 presented a lower hospitalization rate (12.7 vs. 38%, *p* = 0.004), illness duration (18.8 ± 7.7 vs. 29.2 ± 19.5 days, *p* = 0.005), and mortality rate (5.4 vs. 25%, *p* = 0.008). Finally, responders to the vaccination (80% of vaccinated patients) compared with nonresponders showed a reduction in infection duration and hospitalization (5 vs. 40%, *p* = 0.018). In conclusion, we found that COVID-19 presentation and course in hemodialysis patients have improved over time after the implementation of vaccine campaigns. However, due to the evolving nature of the disease, active surveillance is necessary.

## 1. Introduction

Coronavirus disease 2019 (COVID-19) is characterized by a great heterogeneity of clinical presentation and outcomes, ranging from asymptomatic infections to severe forms leading to mortality [1]. Consequently, many studies have evaluated the impact of different risk factors on the COVID-19 course and outcome. These studies found that COVID-19-related mortality was associated with advanced age, male sex, and other conditions, including obesity, hypertension, cardiovascular disease, diabetes mellitus, chronic lung disease, and cancer [2,3,4]. In this regard, patients developing kidney injury disease during COVID-19, or patients with pre-existing chronic kidney disease (CKD), are exposed to high mortality risk [5]. This consideration is also valid for patients undergoing maintenance hemodialysis, who may be considered a distinct subgroup of COVID-19 patients. Indeed, these patients have some peculiarities that may influence disease course, including a high prevalence of comorbidities, frailty, and a specific asset of the immune system. Moreover, logistical factors related to the organization of dialysis facilities may significantly impact the dissemination and evolution of the infection in this setting [6]. These factors may also account for the high short-time mortality rate observed among hemodialysis patients with COVID-19, which ranged between 20 and 30% of affected patients, especially in the surveys performed during the first phases of the pandemic [7]. Due to the high clinical impact, the COVID-19 pandemic has been the object of extensive studies investigating pathogenetic mechanisms, clinical management, and diagnostic and therapeutic strategies [8,9]. These efforts led to the rapid development of vaccination, which has represented a game-changing strategy showing high effectiveness in the prevention of severe disease, hospitalization, and death related to COVID-19 [10]. This is a crucial point when considering hemodialysis patients because it is well known that these patients present an immune dysfunction characterized by chronic systemic inflammation and premature aging of the immune system, which may impair their capacity to respond to the vaccines [11,12]. About COVID-19 vaccination, discordant results have been reported; indeed, although it seems that a high percentage of hemodialysis patients may mount a humoral response after the vaccine, this response may be of short duration [13,14]. Consequently, despite recent advances in fighting against COVID-19, hemodialysis patients still appear fragile and exposed to high risk. Moreover, apart from vaccination, other factors have emerged in the last months that may influence disease course, including the new virus variants and the availability of new therapeutic approaches, such as antiviral drugs and monoclonal antibodies [15]. Yet, there is no clear data on whether these new factors have affected the disease COVID-19 course in hemodialysis patients. Thus, this study aimed to investigate whether any difference exists in clinical presentation, time course, hospitalization, and mortality of COVID-19 patients undergoing dialysis between the first waves of the pandemic in 2020 and the latter waves in late 2021–early 2022 after the full implementation of vaccination campaigns.

## 2. Materials and Methods

For this retrospective study, we recruited three-weekly maintenance hemodialysis patients treated at San Martino, the University Hospital of Genoa, Italy. We enrolled hemodialysis patients with COVID-19 confirmed by positive real-time reverse transcriptase (RT-PCR) assay for severe acute respiratory syndrome coronavirus 2 (SARS-CoV-2) on nasopharyngeal swabs. We compared patients who resulted positive for COVID-19 in two different periods: (a) from March to December 2020 (Group 1) and (b) from September 2021 to February 2022 (Group 2) after the full implementation of vaccination campaigns, i.e., after all patients were offered the possibility to receive at least two doses of the COVID-19 vaccine on a voluntary basis. Notably, during these different periods, our policy regarding the management of case detection was unchanged. Thus, as previously described, we performed nasopharyngeal swabs only in symptomatic patients or in patients with a history of close contact with a COVID-19 case (relatives, other patients, and so on) [16]. Within Group 2, we included both vaccinated patients and patients who voluntarily refused to be vaccinated. The patients who received vaccination were vaccinated with COVID-19 mRNA BNT162b2 (Pfizer-BioNTech).

The study was performed according to the Declaration of Helsinki and was approved by the local ethics committee (Comitato Etico Regionale Liguria, No 135/2020). All participants provided written informed consent before enrollment.

### 2.1. Data Collection

For each patient, we collected: (i) demographics and clinical data, (ii) clinical conditions, general laboratory, and chest X-ray findings at diagnosis, and (iii) data on hospitalization, disease duration, and mortality. Moreover, we also collected data on thrombotic events (including arteriovenous fistula thrombosis, deep vein thrombosis, pulmonary embolism, acute ischemic attack, or stroke) occurring within 30 days from COVID-19 diagnosis.

Laboratory examinations included: serum levels of inflammatory markers, such as high-sensitivity C-reactive protein (hs-CRP), procalcitonin, interleukin-6 (IL-6), ferritin, and lymphocyte count and neutrophil/lymphocyte ratio. IL-6 was determined at the central hospital laboratory by ELISA. To assess the longitudinal evolution of COVID-19, the laboratory data were also evaluated at 7 and 14 days after the diagnosis. In patients of Group 2, we also collected information on COVID-19 vaccination, including the number of doses administrated and serological response to the vaccination. The latter was assessed between the 10th and 20th days after the second dose of the vaccine through the measurement of anti-S-RBD IgG levels, measured by Liaison SARS-CoV-2 trimeric S IgG Assay (Diasorin, Italy) and expressed as binding antibody units (BAU)/m standardized according to the First WHO International Standard for anti-SARS-CoV-2 immunoglobulin [17]. Patients with antibody levels >13 BAU/mL were responders to the vaccination, while patients with undetectable antibodies or levels <13 BAU/mL were considered nonresponders.

### 2.2. Statistical Analysis

Data are presented as mean ± standard deviation (SD) or median with interquartile range (IQR), if not normally distributed (as evaluated by Shapiro Test). Student *t*-test or nonparametric Mann–Whitney test were used to assess the differences between patients affected by COVID-19 in different periods and between responders and nonresponders. Mixed models for repeated measures, followed by Tukey’s multiple comparison tests, were used to evaluate the temporal evolution of laboratory parameters in the different groups. Proportions for categorical variables were compared using Fisher’s exact test. Time-to-event analyses were performed using: (i) Kaplan–Meier method for survival curves estimation and log-rank test to compare them and (ii) univariate Cox regression models in which risk was reported as hazard ratios (HRs) along with their 95% confidence intervals (CIs). Statistical calculations were performed by STATA package, version 14.2 (StataCorp, 4905 Lakeway Drive, College Station, TX 77845 USA). The null hypothesis was rejected if *p*-values < 0.05.

## 3. Results

### 3.1. Patient Characteristics

Group 1 was constituted of 44 hemodialysis patients (69.3 ± 14.6 years, 24 males) with a length of time on dialysis of 67 ± 58.4 months, 16 (36%) with arteriovenous fistula (AVF).

Thirty-four patients (77%) had a previous history of systemic arterial hypertension, 12 (27.3%) diabetes mellitus, and 30 (68.2%) cardiovascular disease, while 4 patients (9%) had a history of transplantation (three kidney, one liver).

Group 2 was constituted of 55 hemodialysis patients (67.4 ± 15.3 years, 39 males) with a dialysis vintage of 39 ± 29 months, 29 (52%) with AVF. A total of 48 patients (87.3%) had a previous history of systemic arterial hypertension, 23 (41.8%) diabetes mellitus, and 22 (40%) cardiovascular disease, while 8 patients (14%) had a history of transplantation (seven kidney, one liver). Overall, six patients (11%) in Group 2 presented a previous history of COVID-19 infection, two of whom had their first COVID-19 diagnosis in 2020 and then were also included in Group 1, while the remaining four had their first infection in 2021. Comparison between the groups showed that patients in Group 2 presented a significantly lower length of time on dialysis, a higher prevalence of diabetes, and a lower prevalence of cardiovascular disease (Table 1). Among the patients in Group 2, 52 (95%) were vaccinated, and 43 of them (83%) had received a third booster dose of vaccination at the time of COVID-19 diagnosis, a percentage in line with that observed in the whole hemodialysis patient population at our center (where, as of February 2022, 265/282 patients, i.e., 94%, were vaccinated). The mean interval from COVID-19 diagnosis to the last vaccination dose was 112 ± 58 days. According to the serum levels of anti-S-RBD IgG available in 49 vaccinated patients from Group 2, 39 patients (80%) were responsive to the second dose of the vaccine. At disease presentation, nonresponders had a significantly lower BMI than responders and reported a high prevalence of history of autoimmune disorders (Table S1).

### 3.2. Clinical Presentation and Radiological Findings

In Group 2, there was a significantly higher number of asymptomatic patients when compared with patients in Group 1 (38% vs. 10%, *p* = 0.002) (Table 2). Initial symptoms were various, with fever, cough, and shortness of breath being the most common ones. However, patients in Group 2 presented a lower incidence of fever, such as dysgeusia, diarrhea, and myalgia. Oxygen saturation and diastolic blood pressure were not different between the two groups, whereas systolic blood pressure was significantly higher in Group 2 (138 ± 25 vs. 124.7 ± 28 mmHg, *p* = 0.018). Basal chest X-ray was requested on clinical indication in 17 patients (38%) from Group 1 and in 6 patients (10.9%) from Group 2 (*p* = 0.001), mostly showing lung consolidations.

### 3.3. Basal Laboratory Characteristics

**Figure 1:**
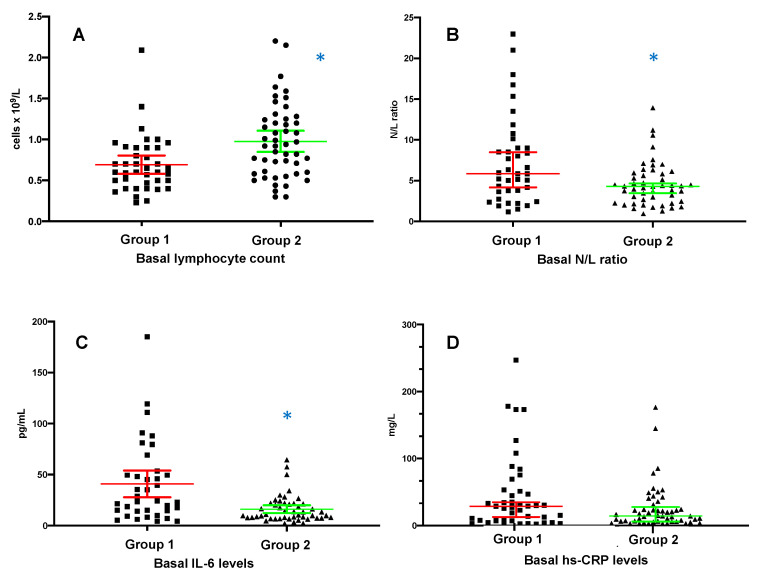
Comparisons of inflammatory parameters between hemodialysis patients affected by COVID-19 during the first pandemic waves of 2020 and last wave of 2021–early 2022. Normally distributed data are expressed as means with 95%CI in (**A**,**C**), while nonnormally distributed data are expressed as medians with 95%CI in (**B**,**D**). * *p* < 0.01. Group 1, from March to December 2020 (red lines); Group 2, from September 2021 to February 2022 (green lines).

Comparison of biochemical parameters collected at diagnosis showed that patients affected by COVID-19 in late 2021, when compared with patients of the 2020 pandemic waves, presented a significantly higher number of lymphocytes (0.97 ± 0.45 vs. 0.69 ± 0.35 cells ×109/L, *p* = 0.008) and lower values of neutrophil/lymphocyte ratio (4.2 (3.5) vs. 5.8 (5.4), *p* = 0.009) and LDH (220 ± 173 vs. 254 ± 98 U/L, *p* = 0.003) (Figure 1). Considering inflammatory markers, patients in Group 2 had comparable levels of procalcitonin and hs-CRP but significantly lower levels of circulating IL-6 (16 ± 13.3 vs. 41 ± 39.4 pg/mL, *p* = 0.002) and ferritin (242 (407) vs. 552.5 (903) μg/L, *p* = 0.021) (Table 3). Patients responsive to the vaccination compared with nonresponders did not show significant differences in basal laboratory parameters (Table S2).

### 3.4. Time Evolution of Inflammatory Parameters

Evaluating the temporal course of the principal laboratory parameters, we found that in Group 1, no significant changes in inflammatory parameters occurred over time. In contrast, in Group 2, we observed a rapid improvement in some parameters such as white blood cell, neutrophil, and lymphocyte count that significantly increased 1 week after diagnosis, accompanied by a significant reduction in hs-CRP levels (14.4 (16.5) at diagnosis vs. 7.4 (20) mg/L at day 7, *p* = 0.002) (Table S3). Serum ferritin levels were not longitudinally collected.

### 3.5. Clinical Outcomes

As for Group 1, 16 patients (38%) were admitted to the hospital because of worsening clinical conditions, while the remaining patients underwent outpatient hemodialysis. No antiviral treatment was prescribed, while 17 patients (63%) received treatment with hydroxychloroquine. Thrombotic events occurred in two patients (4.5%).

Overall, 11 patients died (25%) after a mean of 13 ± 10.5 days from COVID-19 diagnosis, whereas 33 patients resulted negative for SARS-CoV-2 after 29.2 ± 19.5 days from diagnosis. Among Group 2, seven patients (12.7%) were admitted to the hospital. Eight patients (14.5%) were treated with anti-COVID-19 monoclonal antibodies (seven with a combination of casirivimab + imdevimab and one with bamlanivimab + etesevimab), five patients received steroids, and one patient anakinra, an interleukin-1 receptor antagonist. None of the patients in Group 2 was treated with hydroxychloroquine.

**Figure 2:**
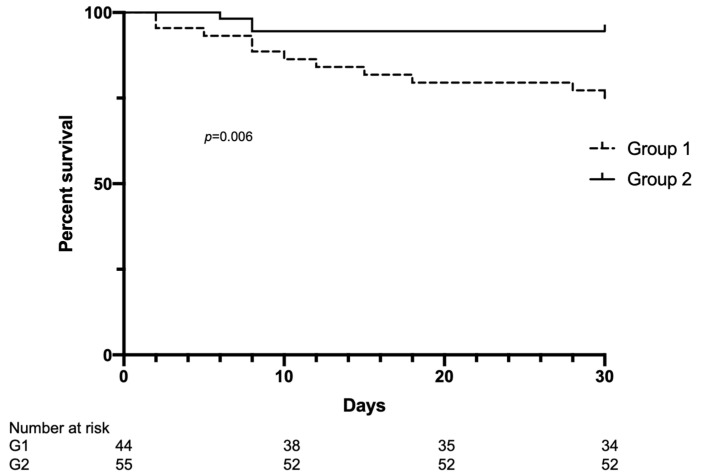
Kaplan–Meier curves of 30-day survival in hemodialysis patients affected by COVID-19 during the first pandemic waves and the last wave of 2021. Group 1, from March to December 2020; Group 2, from September 2021 to February 2022.

**Figure 3:**
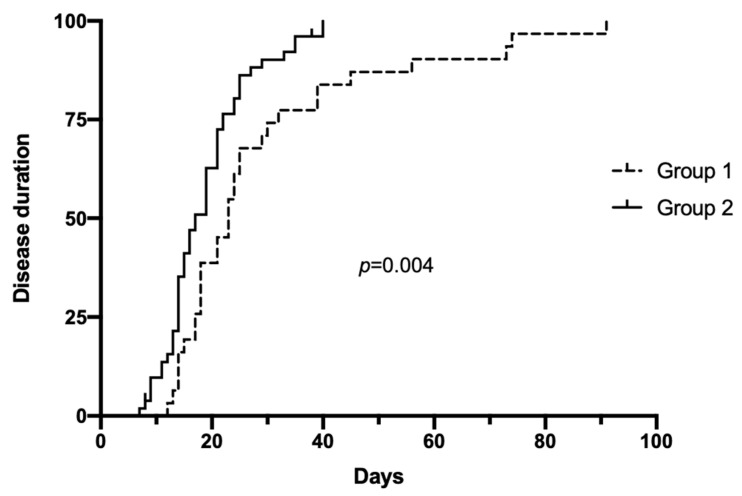
Kaplan–Meier curves for COVID-19 duration (evaluated as time from diagnosis to SARS-CoV-2 PCR negativization) in hemodialysis patients affected by COVID-19 during the first pandemic waves and the last wave of 2021. Group 1, from March to December 2020; Group 2, from September 2021 to February 2022.

Thrombotic events occurred in one patient (1.8%). Overall, three patients died (5.4%) after a mean of 7.3 ± 1.1 days from COVID-19 diagnosis, whereas the remaining 52 patients resulted negative for SARS-CoV-2 after 18.8 ± 7.7 days from diagnosis. Comparison of clinical outcomes showed that Group 2 presented significantly lower mortality, as well as lower hospitalization and infection duration (Table 4, Figure 2 and Figure 3). Among the patients in Group 2, there were no significant differences in clinical outcomes comparing patients treated with anti-COVID-19 monoclonal antibodies or not (infection duration 20 ± 9.5 vs. 17 ± 7.3 days, respectively, *p* = 0.5).

Then, we performed a univariate Cox analysis in the entire cohort of both groups of patients, considering age, gender, length of time on hemodialysis, cardiovascular disease, diabetes, and vaccine as variables. We observed that a history of cardiovascular disease was positively associated with mortality (with HR 1.18, 95%CI 1.005–1.39, *p* = 0.043), whereas vaccination was negatively associated (HR 0.26, 95%CI 0.07–0.96, *p* = 0.04). Multivariate analysis using age, length of time on hemodialysis, and vaccination as covariates confirmed the negative correlation of vaccination with mortality (HR 0.22, 95%CI 0.05–0.91, *p* = 0.037). Finally, comparing responders to the vaccination with nonresponders, we observed that responders presented a significant reduction in infection duration (17.6 ± 6.4 vs. 23.9 ± 7.9 days, *p* = 0.02) and hospitalization rate (5% vs. 40%, *p* = 0.018), while mortality was not different between the two groups (Table S4).

## 4. Discussion

In this study, we evaluated the temporal evolution of the COVID-19 pandemic in hemodialysis patients, comparing patients affected by COVID-19 during the first pandemic waves of 2020 with patients diagnosed with COVID-19 in late 2021–early 2022 after each patient had access to at least two doses of the COVID-19 vaccine. We selected these periods to have the opportunity to assess the clinical impact of full vaccination campaigns and the introduction of specific antiviral treatments developed and diffused during 2021. We observed a significant change in the COVID-19 course in HEMO hemodialysis and, in particular, during the second period, patients presented a milder form of the disease and better clinical outcomes. These patients, compared with those of the first pandemic waves, were more often asymptomatic and reported less frequent fever and specific symptoms, such as myalgia and asthenia. Moreover, they presented higher blood pressure values and less COVID-19-related pulmonary involvement. Significant differences were also found in laboratory examinations that showed in patients from Group 2 a minor degree of lymphopenia and reduced levels of LDH and inflammatory parameters, including ferritin and IL-6 levels.

These findings are relevant because, in COVID-19 patients, the alterations of these parameters have been associated with disease severity and mortality risk [18].

Notably, we observed high intragroup variability in serum ferritin levels that may be the expression of the influence of several confounding factors, including inflammation, epoetin therapy, and iron status. These considerations have led some authors to question the utility of this parameter in hemodialysis patients [19].

Apart from basal measurement, the time course of laboratory examinations was also different between the two groups of patients, with patients from Group 2 showing a significant reduction in inflammatory parameters after a few days from diagnosis.

The better clinical and laboratory profile of patients affected by COVID-19 in late 2021–early 2022 corresponded to a significant improvement in clinical outcomes, depicted as a reduction in infection duration, hospitalization rate, and mortality. Among outcomes, we also considered the occurrence of thrombotic events since early reports showed that COVID-19 in hemodialysis patients is associated with a high risk of thrombosis [20,21]. However, we observed a low rate of thrombotic events in our population without significant differences between the two groups.

On the contrary, the mortality rate observed in our patients during the first phases of the pandemic was in line with that reported in previous studies on hemodialysis patients (25%). Most importantly, we found that mortality decreased in patients affected by COVID-19 in the late phases (5.4%). Overall, our data show that, at least in maintenance hemodialysis patients, COVID-19 has evolved, and disease characteristics and short-term sequelae are changed. It is conceivable that there are many reasons for these changes. First, we noticed some differences in patient characteristics, with patients of the first waves presenting a significantly higher length of time on dialysis, a higher prevalence of cardiovascular disease, and a lower prevalence of diabetes, as compared with the patients from Group 2. Although we found only a weak relationship between a history of cardiovascular disease and death in our cohort, we cannot rule out the possibility that underlying chronic disease may have conditioned COVID-19 expression [22]. It is plausible that a relevant role in the change of COVID-19 presentation and evolution has been played by vaccination. In this regard, we were favored by the active vaccine strategy promoted by the Italian healthcare system leading to the high percentage of vaccinated patients in our cohort. This condition allowed us to observe that, despite initial concerns, also in hemodialysis patients, vaccination was associated with a reduction in mortality and a significant improvement in clinical outcomes [23]. Moreover, we noticed an elevated percentage of patients mounting a sufficient immune response to COVID-19 vaccination, which conceivably further increased after the administration of the third booster dose [24,25]. We found that compared with responsive subjects, the nonresponsive ones presented higher hospitalization and disease duration, while, possibly due to the low sample size, the mortality rate was not different. These data reinforce the idea that vaccination is a game-changing strategy in protecting from severe COVID-19 complications [26]. Moreover, they suggest that, within vaccinated hemodialysis patients, a partial heterogeneity of COVID-19 presentation and course may persist, probably driven by a different underlying immune status. Apart from vaccination, an important aspect to consider when evaluating COVID-19 pandemic evolution is the impact of the new SARS-CoV-2 variants. It is well known that SARS-CoV-2 is a mutating virus, and the emergence of the variants of concern, characterized by different transmissibility and pathogenicity, may affect the disease course [27]. Specifically, in Italy, in the first phases of the pandemic of 2020, the predominant variants were the original Wuhan strain SARS-CoV-2 and B.1.1.7 (Alpha), while in late 2021, B.1.617.2 (Delta) and B.1.1.529 (Omicron) variants were the most prevalent ones [28,29]. The prevalence of different virus variants may explain some differences we found in our cohorts. For example, Omicron replicates faster than all other SARS-CoV-2 variants and has been associated with less dysgeusia and pneumonia [30,31]. Finally, an additional factor potentially contributing to the change in COVID-19 course in hemodialysis patients is the availability of virus-specific treatments, such as monoclonal antibodies or antiviral drugs. These treatments, introduced in 2021, may represent an opportunity to reduce the risk of COVID-19-related hospitalization or death [32]. However, these new therapeutic strategies are still limited in hemodialysis patients, in whom, for instance, the new oral antiviral drugs are contraindicated [33]. Moreover, considering the use of monoclonal antibodies, we found that in our patients infected during the late pandemic wave, the most used antiviral treatment was the combination of casirivimab + imdevimab, which is poorly effective against the Omicron variant [34].

The choice of this treatment was influenced by the fact that at the beginning of 2022, Italy’s health system suffered from a shortage of sotrovimab, a monoclonal antibody active against the Omicron variant [35].

This factor may also explain why we did not find a significant difference in clinical outcomes between patients treated with monoclonal antibodies and untreated patients.

Thus, it seems that the potentialities of COVID-19 treatment have not been fully exploited, while the development of antiviral treatments suitable also for hemodialysis patients seems mandatory. We are aware that the relatively small cohort of studied patients, together with the inability to discriminate and quantify the factors influencing the disease course, including the lack of data on virus variants, are the main weaknesses of this study. Moreover, we have no data on the immune response to the third booster dose of the vaccine that could have reinforced the specific immune response against the infection. To overcome these limitations, there is a need for large prospective longitudinal studies evaluating the time course of both humoral and cellular responses to the vaccination and molecular studies on virus variants’ epidemiology and their clinical impact.

## 5. Conclusions

Our data show that COVID-19 presentation and course in hemodialysis patients have improved over time, especially after the implementation of vaccine campaigns, even if this change was probably a result of the cooperation of different factors rather than the effect of a single element. However, since COVID-19 is a continuing, evolving disease, many questions such as the duration of immunity, the effectiveness of vaccinations, the need for more vaccine doses, the effects of antiviral drugs against new virus variants, and long-term outcomes are still open [36]. Therefore, while continuing the promotion of vaccination and the development of innovative therapeutic strategies remain of paramount importance, active surveillance of the COVID-19 course should be warranted.

## Figures and Tables

**Table 1:** Demographics and medical history at admission of hemodialysis patients affected by COVID-19 during the different periods evaluated.

	Group 1	Group 2	*p*
N	44	55	
Age, yrs	69.3 ± 14.6	67.4 ± 15.3	0.6
Men, *n* (%)	24 (54.5)	39 (70.9)	0.09
BMI, kg/m^2^	24.1 ± 4.9	25 ± 5.2	0.4
Length of time on dialysis, months	67 ± 58.4	39 ± 29	0.015
Vascular access (arteriovenous fistula, *n*%)	16 (36)	29 (52)	0.1
Comorbidities			
Hypertension, *n* (%)	34 (77)	48 (87.2)	0.3
Diabetes mellitus, *n* (%)	12 (27.3)	23 (41.8)	0.03
Cardiovascular disease, *n* (%)	30 (68.2)	22 (40)	0.008
Autoimmune disorders, *n* (%)	1 (2)	5 (9)	0.2
Prior transplant, *n* (%)	4 (9)	8 (14.5)	0.5
History of COVID-19, *n* (%)	-	6 (11)	-
Vaccinated patients, *n* (%)	-	52 (94.5)	-
Vaccinated with booster dose, *n* (%)	-	43 (78.2)	-
Responders after the second dose of vaccine, *n* (%) *	-	39 (80)	

Data are expressed as means ± standard deviations (SD) and were analyzed by Student *t*-test. Proportions for categorical variables were compared using Fisher’s exact test. A *p*-value of <0.05 was considered statistically significant. Abbreviations: coronavirus disease-19, COVID-19; body mass index, BMI. Group 1, from March to December 2020; Group 2, from September 2021 to February 2022. * Data available for 49 patients.

**Table 2:** Clinical presentation and basal radiological findings of hemodialysis patients affected by COVID-19 during the different periods evaluated.

COVID-19 Presentation, *n* (%)	Group 1 (*n*, 44)	Group 2 (*n*, 55)	*p*
Patients symptomatic at diagnosis	40 (90)	35 (62)	0.002
Fever	28 (63.3)	17 (31)	0.002
Cough	17 (38.6)	17 (31)	0.5
Dyspnea (exertional or rest)	14 (32)	4 (7)	0.003
Dysgeusia/anosmia	10 (23)	2 (3.6)	0.005
Fatigue/malaise	3 (6.8)	1 (1.8)	0.8
Gastrointestinal	5 (11.4)	1 (1.8)	0.08
Myalgia	6 (13.6)	0	0.006
Arterial blood oxygen saturation, %	0.95 ± 0.03	0.96 ± 0.03	0.16
Systolic blood pressure, mmHg	124.7 ± 28	138 ± 25	0.018
Diastolic blood pressure, mmHg	62.7 ± 13	67.7 ± 14	0.17
Chest X-ray (clinical indication), *n* (%)	17 (38.6)	6 (10.9)	0.001 *
Lung consolidations	14 (82)	5 (83)	1
Pleural effusion	2 (11.8)	2 (33)	0.2

Data are expressed as means ± standard deviations (SD) and were analyzed by Student *t*-test. Proportions for categorical variables were compared using Fisher’s exact test. A *p*-value of <0.05 was considered statistically significant. Abbreviations: coronavirus disease-19, COVID-19. Group 1, from March to December 2020; Group 2, from September 2021 to February 2022. * The *p*-value for chest X-ray refers to the number of exams performed for clinical indication.

**Table 3:** Basal laboratory parameters of hemodialysis patients affected by COVID-19 during the different periods evaluated.

	Group 1	Group 2	*p*
N	44	55	
WBC count, ×10^9^/L	5.5 ± 3.2	5.5 ± 1.8	0.2
Neutrophil count, ×10^9^/L	4.3 ± 3.2	3.6 ± 1.4	0.5
Neutrophils, (% of WBC)	72.2 ± 17.7	66.7 ± 10.9	0.003
Lymphocyte count, ×10^9^/L	0.69 ± 0.35	0.97 ± 0.45	0.008
Lymphocytes, (% of WBC)	14.7 ± 8.9	18.2 ± 8.1	0.024
Neutrophil/lymphocyte ratio	5.8 (5.4)	4.2 (3.5)	0.009
Platelet count, ×10^9^/L	200 ± 127	194 ± 70	0.3
Hemoglobin, mmol/L	6.7 ± 0.99	6.95 ± 0.8	0.19
Albumin, g/L	34 ± 4	36 ± 7	0.07
LDH, U/L	254 ± 98	220 ± 173	0.003
Procalcitonin, μg/L	1.81 (3.3)	1.40 (1.5)	0.25
hs-CRP, mg/L	28.8 (45.6)	14.4 (16.5)	0.08
Interleukin-6, pg/mL	41 ± 39.4	16 ± 13.3	0.002
Ferritin, μg/L	552.5 (903)	242 (407)	0.02

Normally distributed data are expressed as means ± standard deviations (SD) and were analyzed by Student *t*-test. Nonnormally distributed data are expressed as medians and interquartile ranges (IQR) and were compared by Mann–Whitney test. A *p*-value of <0.05 was considered statistically significant. Abbreviations: coronavirus disease-19, COVID-19; white blood cells, WBC; lactate dehydrogenase, LDH; high-sensitivity C-reactive protein, hs-CRP. Group 1, from March to December 2020; Group 2, from September 2021 to February 2022.

**Table 4:** Clinical outcomes of hemodialysis patients affected by COVID-19 during the different periods evaluated.

	Group 1	Group 2	*p*
N	44	55	
Illness duration, days *	29.2 ± 19.5	18.8 ± 7.7	0.005
Thrombotic events, *n* (%)	2 (4.5)	1 (1.8)	0.5
Hospitalized patients, *n* (%)	17 (38)	7 (12.7)	0.004
Deaths, *n* (%)	11 (25)	3 (5.4)	0.008
Time from diagnosis to death, days	13 ± 10.5	7.3 ± 1.1	0.4

Data are expressed as means ± standard deviations (SD) and were analyzed by Student *t*-test. Proportions for categorical variables were compared using Fisher’s exact test. A *p*-value of <0.05 was considered statistically significant. * Illness duration was calculated only in survivors from the date of the first positive RT-PCR assay for SARS-CoV-2 infection to the date of the two consecutive negative RT-PCR assays. Group 1, from March to December 2020; Group 2, from September 2021 to February 2022.

## Data Availability

Data will be made available on reasonable request from the corresponding author.

## Supplementary Materials

The following supporting information can be downloaded at: https://www.mdpi.com/article/10.3390/ijerph191710836/s1, Table S1: demographics and medical history at admission of hemodialysis patients affected by COVID-19 according to the response to the second dose of COVID-19 mRNA vaccine, Table S2: basal laboratory parameters of hemodialysis patients affected by COVID-19 according to the response to the second dose of COVID-19 mRNA vaccine, Table S3: time course of laboratory parameters in hemodialysis patients affected by COVID-19 during different periods, Table S4: clinical outcomes of hemodialysis patients affected by COVID-19 according to the response to the second dose of COVID-19 mRNA vaccine.
