# Supplementary material for: The Evolving Scenario of COVID-19 in Hemodialysis Patients
Source: Int J Environ Res Public Health. 2022 Aug 31;19(17):10836. doi: 10.3390/ijerph191710836 (PMC9518574; doi:10.3390/ijerph191710836)
Supplement: Supplementary file 1 [file ijerph-19-10836-s001.zip › ijerph-1830954-supplementary.pdf]

The Evolving Scenario of COVID-19 in Hemodialysis Patients

Supplementary Materials

Table S1. Demographics and medical history at admission of hemodialysis patients affected by COVID-19 according to the response to the second dose of COVID-19 mRNA vaccine.

	Responders	Non-responders	*p*
n, *	39	10	
Age, yr	67.7±18	68.4±17	0.9
Men, n (%)	30 (76)	8 (80)	1
BMI, kg/m^2	26±5.2	22.2±4.5	0.02
Length of time on dialysis, months	39±30.5	45±22	0.27
Comorbidities			
Hypertension, n (%)	35 (89)	7 (70)	0.1
Diabetes mellitus, n (%)	17 (43)	4 (40)	1
Cardiovascular disease, n (%)	12 (30)	6 (60)	0.1
Autoimmune disorders, n (%)	2 (5)	3 (30)	0.05
Prior transplant, n (%)	4 (10)	1 (10)	1
History of COVID-19, n (%)	4 (10)	0	0.5
Vaccinated with booster dose, n (%)	33 (84)	8 (80)	0.6

Data are expressed as means ± standard deviations (SD) and were analyzed by Student T-test. Proportions for categorical variables were compared using Fisher's exact test. A P-value <0.05 was considered statistically significant. Abbreviations: Coronavirus disease-19, COVID-19; body mass index, BMI.
*data on anti-S-RBD IgG levels were available for 49 patients

Table S2. Basal laboratory parameters of hemodialysis patients affected by COVID-19 according to the response to the second dose of COVID-19 mRNA vaccine.

	Responders	Non-responders	*p*
WBC Count, ×10^9/L	5.6 ± 1.7	5.7 ± 2.1	0.8
Neutrophil count, ×10^9/L	4 ± 1.5	3.6 ± 1.5	0.4
Neutrophil %	73 ± 10	65 ± 10.8	0.037
Lymphocyte count ,×10^9/L	0.97 ± 0.3	0.88 ± 0.6	0.18
Lymphocyte %	15.1 ± 9	18.6 ± 7.8	0.12
Neutrophil/lymphocyte ratio	4.3 (2.6)	5.9 (6.5)	0.15
Albumin, g/L	37 ± 4	33 ± 6	0.15
LDH, U/L	234 ± 206	192 ± 32	0.4
Procalcitonin, μg/L	1.5 (1.8)	1 (0.7)	0.4
hs-CRP, mg/L	13 (27.5)	15.8 (14.5)	0.9
Interleukin-6, pg/mL	14.8 ± 10	16.3 ± 17.8	0.7
Ferritin, μg/L	228 (903)	216 (407)	0.08

Normally distributed data are expressed as means ± standard deviations (SD) and were analyzed by Student T-test. Non-normally distributed data are expressed as medians and interquartile ranges (IQR) and were compared by Mann-Whitney test. A P-value <0.05 was considered statistically significant.
Abbreviations: Coronavirus disease-19, COVID-19; white blood cells, WBC; lactate dehydrogenase, LDH; high-sensitivity C-reactive protein, hs-CRP.
Group 1, from March to December 2020; Group 2, from September 2021 to February 2022

Table S3. Time course of laboratory parameters in hemodialysis patients affected by COVID-19 during different periods.

	**Group 1**			*p*	**Group 2**			*p*
	**Day 0**	**Day 7**	**Day 14**		**Day 0**	**Day 7**	**Day 14**	
WBC Count, $\times 10^9$/L	5.5 ± 3.2	5.6 ± 2.1	5.7 ± 2.4	0.2	5.5 ± 1.8	6.6 ± 1.7	6.4 ± 2	<0.001
Neutrophil count, $\times 10^9$/L	4.3 ± 3.2	4.4 ± 2	4.1 ± 2	0.14	3.6 ± 1.4	4.4 ± 1.4	4.4 ± 1.6	0.004
Lymphocyte count, $\times 10^9$/L	0.69 ± 0.35	0.74 ± 0.36	0.77 ± 0.3	0.6	0.97 ± 0.45	1.2 ± 0.5	1.2 ± 0.48	<0.001
Neutrophil/lymphocyte ratio	5.8 (5.4)	4.9 (3.2)	4.6 (4.1)	0.9	4.2 (3.5)	4 (3.3)	4 (3.2)	0.7
LDH, U/L	254 ± 98	221 ± 59	236 ± 95	0.5	220 ± 173	200 ± 86	198 ± 30	0.7
hs-CRP, mg/L	28.8 (45.6)	12.7 (18.7)	16.3(26.8)	0.08	14.4 (16.5)	7.4 (20)	5 (14)	0.002

Normally distributed data are expressed as means ± standard deviations (SD) and non-normally distributed data are expressed as medians and interquartile ranges (IQR). Comparisons were made using mixed models for repeated measures, followed by Tukey's multiple comparison tests. A P-value <0.05 was considered statistically significant.

Abbreviations: Coronavirus disease-19, COVID-19; white blood cells, WBC; lactate dehydrogenase, LDH; high-sensitivity C-reactive protein, hs-CRP.

Group 1, from March to December 2020; Group 2, from September 2021 to February 2022

Table S4. Clinical outcomes of hemodialysis patients affected by COVID-19 according to the response to the second dose of COVID-19 mRNA vaccine.

	**Responders**	**Non-Responders**	*p*
n,	39	10	
Illness duration, days *	17.6 ± 6.4	23.9±7.9	0.02
Hospitalized patients, n (%) +	2 (5)	4 (40)	0.018
Deaths, n (%)	2 (5)	1 (10)	0.5

Data are expressed as means ± standard deviations (SD) and were analyzed by Student T-test. Proportions for categorical variables were compared using Fisher's exact test. A P-value <0.05 was considered statistically significant.

* Illness duration was calculated only in survivors from the date of the first positive RT-PCR assay for SARS-COV-2 infection to the date of the two consecutive negative RT-PCR assays.

+ 1 additional hospitalization occurred in an unvaccinated patient
